# *De Novo* Whole-Genome Sequence and Annotation of a *Leishmania* Strain Isolated from a Case of Post-Kala-Azar Dermal Leishmaniasis

**DOI:** 10.1128/genomeA.00809-15

**Published:** 2015-07-16

**Authors:** Anil Kumar Gupta, Saumya Srivastava, Amit Singh, Sarman Singh

**Affiliations:** Division of Clinical Microbiology & Molecular Medicine, Department of Laboratory Medicine, All India Institute of Medical Sciences, New Delhi, India

## Abstract

The pathogenesis of post-kala-azar dermal leishmaniasis (PKDL) is complex. Only 5 to 10% of kala-azar patients develop this dermal complication, and it is not known whether this is due to changes in the parasite genome or some host factors. Here, we report the whole-genome sequence and annotated genes of the whole genome of the PKDL strain.

## GENOME ANNOUNCEMENT

Post-kala-azar dermal leishmaniasis (PKDL) is a well-known dermal sequela of visceral leishmaniasis (VL) or kala-azar. PKDL manifests as maculopapular skin lesions several months or years after the cure of VL, but only in 5 to 10% of patients. Our group has been of the opinion that PKDL is a result of *in vivo* generation of quasi-species of *Leishmania donovani* either as *in vivo* hybridization of various endemically circulating species within the host cells or due to superinfection with other organisms (1). Therefore, this study was undertaken to sequence the genome of the *Leishmania* isolated from the skin lesion of a PKDL patient.

The whole-genome sequencing was performed using 2 × 100-bp paired-end reads on an Illumina HiSeq 2500 system, which produced 36.3 million reads, corresponding to more than 110-fold sequencing depth (2). Low-quality and adopter sequences were trimmed from the reads and filtered out depending upon phred quality score (*Q* score) of individual base (3). *De novo* assembly of reads was constructed to longer scaffolds using the A5 assembly pipeline. The draft genome of the *Leishmania* strain consisted of 1,100 sequence scaffolds (minimum, 502 bp; maximum, 265,960 bp; *N*_50_, 61,709 bp) with a total length of 27,848,322 bp (27.8 Mbp) and an average G+C content of 55.8%. CEGMA v2.4 was used to remove short segmental hits from the contigs and 12 contigs (29 kbp) were identified, showing homology with kinetoplast DNA of *Leishmania*. Pairwise alignment was performed with *Leishmania donovani* Ld2001 (4), which showed 6,712 variable sites (single-nucleotide polymorphisms [SNPs] and insertion and deletions [indels]) in the genome of our PKDL isolate with 744 synonymous, 1,068 nonsynonymous SNPs, and 20 frameshift indels. Using Tandem Repeat Finder (5) we found 28,800 tandem repeats in the entire genome and >5,758 (20%) were in the coding gene regions. Overall, 8,336 protein coding genes were predicted, in the genome of which 8,092 (97.1%) genes had complete gene sequences with mean length of 1,746 bp. Additionally, 7,876 orthologous genes were predicted, and 84.3% of these were coding more than one protein. The gene coding for glycoprotein gp63 was identified as the largest orthologous gene family.

At least 138 tRNAs were identified in the genome. This number is significantly higher than that of other *Leishmania* isolates sequenced so far. On sequence mapping, 4.2% reads were mapped with *L. infantum*, 4.6% with *L. major*, and 1.8% with *L. braziliensis*. While mapping the PKDL contigs with three Indian VL isolates (4), we found 98.3%, 88.2%, and 70.3% coverage with the genomes of *L. donovani* Ld2001, *L. donovani* Ld39, and *L. donovani* BHU1095 strains, respectively*.* The genome of our isolate showed >98.0% sequence homology to all the three Indian strains. The BLAST analysis suggested that the 11,281 sequence reads had homology with *Leptomonas seymouri* (possibly coinfection) as reported earlier also but we also found 893 contigs of a heterotrophic bacterium *Parvibaculum lavamentivorans* DS-1 in the sequencing data, which covers 53.8% of the *Parvibaculum lavamentivorans* DS-1 genome. This indicates a possibility of endosymbiotic infection/superinfection of the *Leishmania* leading to PKDL manifestations.

### Nucleotide sequence accession number.

The whole-genome sequence described here has been deposited at GenBank under the accession no. LBGS00000000.
